# Simultaneous targeted activation of Notch1 and *Vhl*-disruption in the kidney proximal epithelial tubular cells in mice

**DOI:** 10.1038/srep30739

**Published:** 2016-08-05

**Authors:** Elinn Johansson, Birgitte Rönö, Martin Johansson, David Lindgren, Christina Möller, Håkan Axelson, Emma M. K. Smith

**Affiliations:** 1 Division of Translational Cancer Research, Department of Laboratory Medicine, Medicon Village, Building 404 A3, Scheelevägen 8, 404A3, 223 63 Lund, Sweden; 2 Center for Molecular Pathology, Department of Translational Medicine, Skåne University Hospital, 205 02 Malmö, Sweden

## Abstract

Clear cell renal cell carcinoma (ccRCC) is the most common subtype of kidney cancer, representing approximately 75% of all renal neoplasms. ccRCC is known to be strongly associated with silencing of the *von Hippel Lindau* (*VHL*) tumor suppressor gene, yet *VHL* deficiency alone does not seem to be sufficient to drive the oncogenic transformation of normal renal epithelium and induce renal tumorigenesis. We, and others, have previously suggested that constitutive activation of the Notch signaling pathway, alongside with *VHL* loss, contribute to the oncogenic features of ccRCC. Here we report a prevailing hyperactivation of the Notch1 receptor in human ccRCC relative to the healthy counterpart. To explore the consequences of the elevated Notch1 signaling observed in ccRCC patient material, we made use of a conditional mouse model based on concurrent ectopic expression of constitutively active Notch1 (NICD1) and deletion of the *Vhl* gene. Histological examination of the kidneys of the conditional mice demonstrate the existence of nests of dysplastic cells with a clear cytoplasm as a consequence of lipid accumulation, thus displaying a one important hallmark of human ccRCC.

Renal cancers comprise a diverse group of solid tumors that account for approximately 3% of all new cancer cases each year^1^. Clear cell renal cell carcinoma (ccRCC) is by far the most common renal neoplasm representing about 75% of all cases^2^,^3^. Several lines of evidence indicate that ccRCC tumors originate from the proximal tubular compartment^4^,^5^. Histologically, ccRCC is characterized by solid nests of tumor cells with a clear cytoplasm, which is due to an abnormal cytoplasmatic accumulation of cholesterol, cholesterol esters, other neutral lipids and glycogen^6^,^7^. The vast majority of sporadic ccRCCs are associated with somatic biallelic inactivation of the tumor suppressor gene *von Hippel-Lindau* (*VHL*) and the consequent stabilization of hypoxia-inducible factor (HIF) α subunits, and hence a constitutively activated hypoxic response^8^,^9^,^10^. Although the absence of functional VHL protein (pVHL) and the resulting accumulation of HIF-α is crucial to ccRCC pathogenesis^11^,^12^,^13^, kidney-restricted ablation of *Vhl* in transgenic mice has repeatedly been shown to be insufficient to induce renal tumorigenesis^14^,^15^,^16^,^17^. Moreover, germline inactivation of the *VHL* gene, associated with the von Hippel-Lindau syndrome, is accompanied by a high frequency of renal cysts, which only occasionally develop into ccRCC^18^. Taken together, these observations strongly indicate that in addition to *VHL*-inactivation, there is a requirement for additional genetic alterations to provoke ccRCC tumor development. This statement was further supported by recent major sequencing efforts that indicate that chromatin modifying genes often are mutated in ccRCC in conjunction with loss of pVHL, thus representing a major cooperating pathway^19^,^20^,^21^.

Notch signaling is an evolutionary conserved pathway of great importance both during embryonic development and postnatal life. Aberrant Notch signaling has been associated with multiple human disorders, particularly a wide range of cancers in which Notch, depending on the affected tissue and cell type, act as a tumor suppressor or, when overactive, a potent oncogene^22^. The oncogenic role of Notch was first established in 1991 in T-cell acute lymphoblastic leukemia (T-ALL)^23^, and has since then gradually expanded to include also breast cancer^24^,^25^, lung adenocarcinoma^26^,^27^, melanoma^28^, and glioma^29^ to name a few. The consequences of Notch-driven tumorigenesis can partly be explained by the function of direct target genes as inducers of proliferation and tumor-cell survival^30^. Recent evidence has suggested that Notch signaling also regulates self-renewal and survival of cancer stem cells (CSCs)^31^,^32^, participates in epithelial-mesenchymal transition (EMT)^33^, increases therapy resistance^34^,^35^,^36^, triggers a metabolic switch towards aerobic glycolysis, also known as the Warburg effect^37^, and affects tumor angiogenesis^38^,^39^.

During the past years Notch has emerged as a potential oncogene also in renal neoplasms; several research groups have concluded that high expression of the Notch ligands Jagged1 and Delta-like4 correlates with an increased risk of distant metastasis and worse prognosis in patients with ccRCC^40^,^41^,^42^.

Moreover, we have previously shown that the expression level of the Notch1 receptor is significantly increased in ccRCC cells compared with the adjacent non-neoplastic renal tissue, and that blocking of Notch1 attenuates the proliferative growth of ccRCC cell lines and primary cultures *in vitro* and in a xenograft model *in vivo*^43^. Notch signaling has also been reported to stimulate the invasive and migratory capacity of ccRCC cells^41^,^44^,^45^,^46^ and to have a direct role in the development of EMT in cultured human primary proximal epithelial cells^47^.

To determine whether Notch signaling could induce an oncogenic switch in renal proximal tubular epithelial cells (PTECs) *in vivo*, we used a conditional mouse model based on the ectopic expression of constitutively active intracellular domain of Notch1 (NICD1) and the disruption of the *Vhl*-gene. These mice did not develop any apparent renal disorder during the time course of this study. However, immunohistological analysis of the kidneys of the conditional mice revealed nests of dysplatic cells with a clear cytoplasm as a consequence of lipid accumulation, thus reproducing the key feature of early human ccRCC carcinogenesis.

## Results

### Increased activity of Notch1 signaling in ccRCC

**Figure 1:**
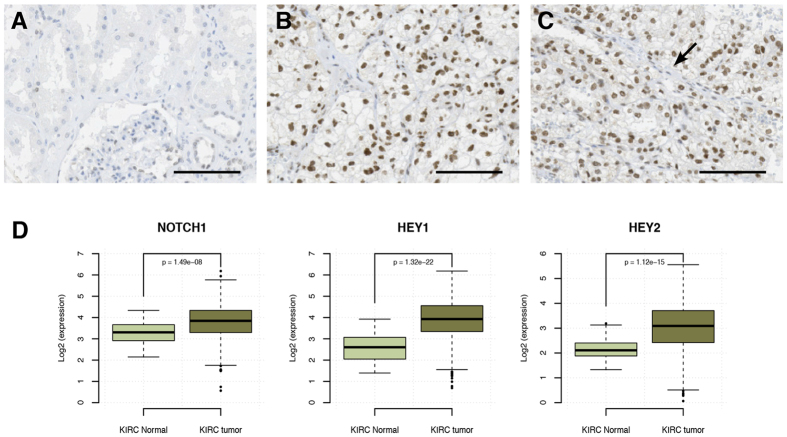
Notch1 is hyperactivated in ccRCC diseased tissue. Representative pictures of (**A**) normal kidney and (**B,C**) ccRCC from a TMA stained with an antibody directed against human NICD1. Arrow indicates NICD1 negative stromal tissue border. Scale bars, 100 μm. (**D**) Relative RNAseq gene expression levels of *NOTCH1* and the Notch target genes *HEY1* and *HEY2* in the TCGA data comprising 70 normal kidney tissue samples and 530 ccRCCs. For statistical analysis a Wilcocon test was performed.

We have previously reported that the Notch1 receptor is upregulated in human ccRCC compared to healthy renal epithelial cells^43^. To examine whether Notch1 activity, i.e. signaling through the Notch1 receptor, also was elevated in ccRCC, we stained a tissue micro array (TMA) with 56 ccRCC samples and 4 samples with normal renal tissue, using an antibody raised against the intracellular part of cleaved human Notch1 (NICD1) (Fig. 1A–C). We found positive staining in 53 out of 56 tumors, whereas the normal renal tissue showed no NICD1 staining (Fig. 1A). It was also clear that the activation of Notch1 was restricted to the tumor compartment as the surrounding stromal tissue stained negatively for NICD1 (Fig. 1C, arrow). Furthermore, analyzes of the TCGA data set comprising 527 ccRCC and 128 normal kidney cortical samples revealed elevated expression of Notch1 and the Notch target genes Hey1 and Hey2 in ccRCC in comparison to the normal samples (Fig. 1D,E). These results corroborate previous reports showing that elevated Notch1 signaling is common feature of human ccRCC^43^,^44^,^48^.

### The ectopic expression of NICD1 and silencing of *Vhl* is restricted to the PTECs in androgen treated *Vhl* ^*fl/fl*^/*CALSL-NICD/Kap2-iCre* transgenic mice

**Figure 2:**
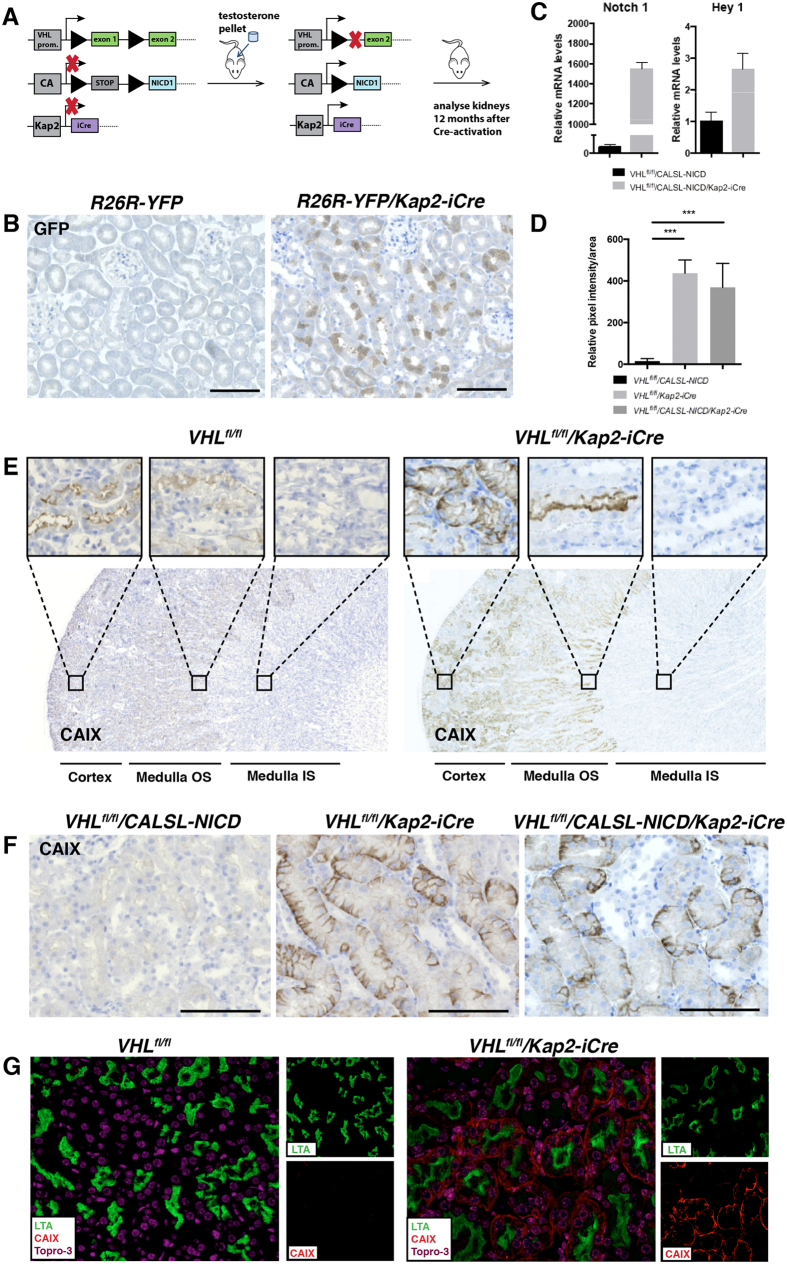
(**A**) Schematic drawing illustrating the transgenic mice used in this project. In order to induce Cre-mediated ectopic expression of NICD and/or conditional inactivation of *Vhl* in the epithelial tubular cells (mPTEC) *in vivo*, we crossed *Vhl*^*fl/fl*^ and CALSL-*NICD* transgenic mice with the mPTEC specific androgen-inducible *Kap2-iCre* transgene, giving *Vhl*^*fl/fl*^*/CALSL-NICD/Kap2-iCre* mice. loxP sites are represented by triangles. CA – chicken β-actin promoter. (**B**) YFP staining 30 days after androgen treatment of *R26R-YFP* and *R26R-YFP/Kap2-iCre* mice showing proximal tubule specific staining. (**C**) Up regulation of *Notch1* and *Hey1* mRNA in kidneys 12 months after androgen treatment of *Vhl*^*fl/fl*^*/CALSL-NICD/Kap2-iCre* mice compared to control mice. (**D**) Quantification of CAIX expression defined as pixel intensity per area in androgen treated mice with various genotypes. Asterisks indicate statistical significance ***p < 0.001, **p < 0.01. (**E**) CAIX staining 12 months after androgen treatment of control and *Vhl*^*fl/fl*^*/Kap2-iCre* mice. Specific basolateral CAIX staining was only detected in the renal cortex of *VHL*^*fl/fl*^*/Kap2-iCre* mice, but not in the outer stripe (OS) or inner stripe (IS) of the medulla. (f) CAIX staining of FFPE kidney sections from *Vhl*^*fl/fl*^*/CALSL-NICD, Vhl*^*fl/fl*^*/Kap2-iCre* and *Vhl*^*fl/fl*^*/CALSL-NICD/Kap2-iCre* mice 12 months after onset of androgen treatment (n = 12 in each group). (**G**) IF co-staining of CAIX (red) and LTA (green) in androgen treated *Vhl*^*fl/fl*^ and *Vhl*^*fl/fl*^*/Kap2-iCre* mice. Scale bars 100 μm.

Currently, existing data supports a role for Notch1 in the tumorigenic process of ccRCC^43^,^45^,^48^,^49^,^50^. To test whether Notch1 signaling act as a key factor in ccRCC-development *in vivo*, we crossed the transgenic mouse line *CALSL-NICD* that conditionally confers ectopic expression of human *NICD1*^51^, with mice carrying a floxed *Vhl* (*Vhl*^*fl/fl*^) allele^52^, and the *Kap2-iCre* mouse strain^53^, in which improved *Cre* (*iCre*) is driven by the androgen inducible and PTEC specific kidney androgen protein 2 (*Kap2*) promoter (Fig. 2A). Our choice of *Cre*-driver in this system was based on recent data that reinforces the notion that ccRCC originates from the proximal compartment of the nephron^4^,^5^, and the fact that *VHL* loss in conjunction with development of sporadic ccRCC most probably occurs in fully differentiated adult proximal tubular cells. To assure that the *Kap2*-promoter is androgen inducible, and its activity is limited to the renal PTECs, we crossed the *Kap2-iCre* transgenic mouse with an *R26R-YFP* reporter mouse. Upon immunohistological analysis of the *R26R-YFP*/*Kap2-iCre* mice, focal YFP expression was detected in a subset of tubules in the renal cortex of the androgen treated *R26R-YFP*/*Kap2-iCre* animals but not in the control group (Fig. 2B).

Next, we wanted to verify adequate *Kap2-iCre* controlled activation/excision of the *CALSL-NICD* and *Vhl*^*fl/fl*^ transgenes. For this purpose, we quantified the expression levels of *Notch1* and the Notch target gene *Hey1* in renal cortex of *Vhl*^*fl/fl*^/*CALSL-NICD/Kap2-iCre* by qPCR. As expected, androgen treatment significantly enhanced the expression of *Notch1* and *Hey1* in *Vhl*^*fl/fl*^/*CALSL-NICD/Kap2-iCre* mice compared to control (Fig. 2C).

**Figure 3:**
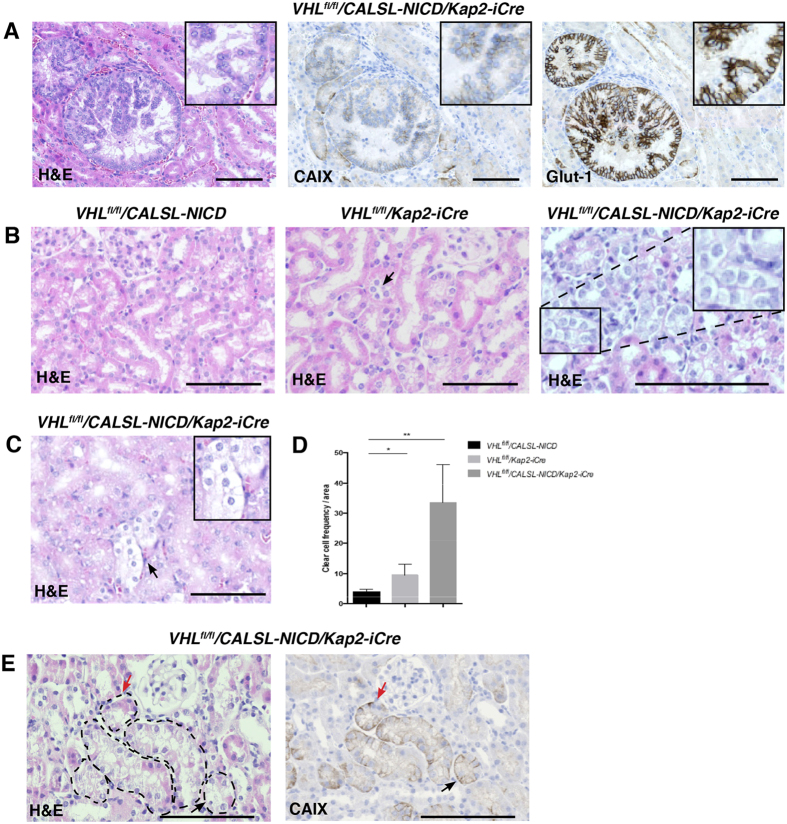
Ectopic activation of NICD1 is associated with the appearance of cells with a clear cytoplasm in the renal cortex. (**A**) H&E staining of a neoplastic lesion with papillary growth pattern and clear signs of cellular dysplasia found in a *VHL*^*fl/fl*^*/CALSL-NICD/Kap2-iCre* mice 9 months post androgen induction. The tumor stained positive for the VHL downstream targets CAIX (middle) and Glut-1 (right). (**B**) Representative pictures (n = 12) of H&E staining of FFPE kidney sections from *Vhl*^*fl/fl*^*/CALSL-NICD, Vhl*^*fl/fl*^*/Kap2-iCre* and *Vhl*^*fl/fl*^*/CALSL-NICD/Kap2-iCre* 12 months after androgen induction. (**C**) Representative picture of androgen treated *VHL*^*fl/fl*^*/CALSL-NICD/Kap2-iCre* mice with “clear cell” phenotype. Scale bars, 100 μm. (**D**) Quantification of clear cells per area unit in androgen treated mice with various genotypes. Asterisks indicate statistical significance **p < 0.01, *p < 0.05. (**E**) Representative picture of consecutive sections from androgen treated *VHL*^*fl/fl*^*/CALSL-NICD/Kap2-iCre* mouse. The cells presented with a clear cytoplasm in H&E staining (left panel, black arrow), coincide with *VHL* loss indicated by strong CaIX staining (right panel black arrow), whereas absence of clear cytoplasm (red arrows) coincide with lack of CAIX staining.

Carbonic anhydrase IX (CAIX) is a well-accepted surrogate marker of hypoxia that is known to be up-regulated upon loss of *Vhl*^54^. In accordance with this, CAIX staining was detected at similar levels in the kidney cortex of androgen treated *Vhl*^*fl/fl*^/*CALSL-NICD/Kap2-iCre* and *Vhl*^*fl/fl*^/*Kap2-iCre* mice, but not in control mice (Fig. 2D–F). Immunofluorescent co-staining of CAIX and the PTEC marker Lotus tetragonolobus agglutinin (LTA) confirmed that the *Vhl* was deleted specifically in the proximal tubules (Fig. 3G). Taken together, these results indicate that the *Kap2-iCre* transgene admits to efficient Cre-mediated recombination of the *CALSL-NICD* and *Vhl*^*fl/fl*^ transgenes in an androgen dependent and PTEC-restricted manner.

### Ectopic expression of constitutively active Notch1 drives the formation of dysplastic PTECs with a clear cytoplasm

To evaluate the consequences of ectopic expression of NICD1 and/or the loss of *Vhl* in the PTECs, *Vhl*^*fl/fl*^*/CALSL-NICD/Kap2-iCre* and *Vhl*^*fl/fl*^*/Kap2-iCre* were sacrificed 12 months after receiving a subcutaneous implant of a 10 mg, thirty-day release testosterone pellet.

The androgen treated animals appeared healthy all through the experiment, and no prominent difference in the renal gross morphology between androgen treated animals and the respective control group, or between androgen induced *Vhl*^*fl/fl*^*/CALSL-NICD/Kap2-iCre* and *Vhl*^*fl/fl*^*/Kap2-iCre* mice, was noted at the time of analysis. To asses differences at the molecular level; we first stained formalin fixed paraffin embedded (FFPE) kidneys from androgen treated *Vhl*^*fl/fl*^*/CALSL-NICD/Kap2-iCre*, *Vhl*^*fl/fl*^*/Kap2-iCre* and *Vhl*^*fl/fl*^*/CALSL-NICD* mice with the proliferation marker ki67. In this mouse model we noticed no difference in tubular or interstitial proliferation index as shown by ki67 staining (Figure S1A). It has been reported that *Vhl* deletion might lead to increased renal fibrosis^55^. To investigate if this was the case also in our model, we stained the kidney tissue from the androgen induced *Vhl*^*fl/fl*^*/CALSL-NICD/Kap2-iCre*, *Vhl*^*fl/fl*^*/Kap2-iCre* and *Vhl*^*fl/fl*^*/CALSL-NICD* mice with Masson’s Trichrome histochemical stain, in order to assess changes in the extent of kidney fibrosis. However, we noted no significant increase in the degree of interstitial or tubular fibrosis, indicating that fibrosis is not affected in our model system (Figure S1B). Further, podocalyxin staining showed that silencing of *Vhl* and ectopic expression of NICD1 did not confer any changes in the renal vascular structure (Figure S1C).

With regards to malignant growth, we found a lesion in one of the androgen induced *Vhl*^*fl/fl*^*/CALSL-NICD/Kap2-iCre* mice (n = 12) (Fig. 3). Histologically it consisted of a thin surrounding pseudocapsule covering a tumor displaying a papillary and cribriform growth pattern. The tumor cells showed distinct nuclear pleomorphismhad and slightly enlarged nuclei. To verify that this adenoma was indeed associated with *Vhl* deletion, we stained consecutive sections of the lesion with antibodies directed against acknowledged HIF target proteins as CAIX (Fig. 3, middle) and Glut-1 (Fig. 3, right). These markers are also used clinically as histopathological markers for ccRCC. The tumor stained positively for both markers indicating that this lesion displayed the characteristics of *Vhl* deletion.

**Figure 4:**
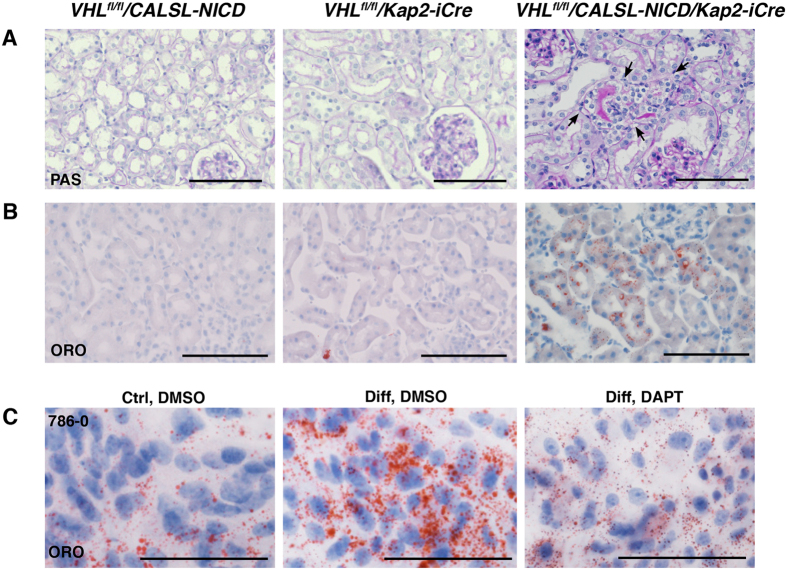
The clear cell phenotype is linked to the accumulation of cytoplasmic lipids. (**A**) Representative pictures of Periodic Acid Schiff (PAS) staining of kidney sections from androgen treated *Vhl*^*fl/fl*^*/CALSL-NICD/Kap2-iCre*, *Vhl*^*fl/fl*^*/Kap2-iCre* or *Vhl*^*fl/fl*^*/CALSL-NICD* mice. No cytoplasmic accumulation of glycogen was observed in any of the monitored mice. *Vhl*^*fl/fl*^*/CALSL-NICD/Kap2-iCre* mice however displayed small nests disrupted tubular structure (arrows) that was not present in control mice. (**B**) ORO staining demonstrates accumulation of neutral lipids in androgen treated *VHL*^*fl/fl*^*/CALSL-NICD/Kap2-iCre* mice, but not in *Vhl*^*fl/fl*^*/Kap2-iCre* or *Vhl*^*fl/fl*^*/CALSL-NICD* mice. (**C**) ORO staining of 786–0 cells cultured in conventional growth medium (left), or adipocyte differentiation medium (middle and right). Cells were treated with DAPT (right) or DMSO (ctrl). Scale bars, 100 μm.

Surprisingly, upon H&E staining of androgen treated *Vhl*^*fl/fl*^*/CALSL-NICD/Kap2-iCre* mice frequently displayed nests of dysplastic cells with a clear cytoplasm (Fig. 3B,C). Quantification of the number of areas with clear cells per area, demonstrates that the number of these cells was significantly increased in the androgen treated *Vhl*^*fl/fl*^*/CALSL-NICD/Kap2-iCre* mice compared to the androgen treated *Vhl*^*fl/fl*^*/Kap2-iCre* mice (Fig. 3D). H&E and CAIX staining on consecutive sections from *Vhl*^*fl/fl*^*/CALSL-NICD/Kap2-iCre* mice showed co-concordance of the clear cell phenotype with markers for *Vhl* loss (Fig. 4E). Together, this data suggest that the clear cell phenotype is induced by the activation of NICD1 in the context of *Vhl* deletion.

### The Notch signaling pathway plays a role in renal lipidogenesis

As the clear cell morphology is hallmark of ccRCC, we wanted to further analyze the cellular content of the clear cells observed in the androgen treated *Vhl*^*fl/fl*^*/CALSL-NICD/Kap2-iCre* mice. We could however not detect any increase in staining with Periodic acid-Schiff (PAS) in the clear cell nodules, indicating that the clear cell morphology was not a consequence of increased deposition of glycogen (Fig. 4A).

Next, we performed Oil red O (ORO) staining to assess accumulation of triglycerides and other neutral lipids. Interestingly, ORO staining was only detected in the androgen treated *Vhl*^*fl/fl*^*/CALSL-NICD/Kap2-iCre* mice, but not in the androgen treated *Vhl*^*fl/fl*^*/Kap2-iCre* mice (Fig. 4B). It should be noted that the ORO stainings were performed using cryopreserved tissue, which explains the lack of cytoplasmic clearing associated with FFPE tissue processing. These results suggest that activation of Notch plays a role in the lipid accumulation process that occurs in the *VHL* negative proximal tubular cells during ccRCC progression.

To further substantiate a role for Notch signaling in accumulation of lipids in ccRCC cells, we performed *in vitro* experiments using an established ccRCC cell line 786-O. 786-O cells, like virtually all established ccRCC cell lines, display a sarcomatoid morphology during conventional *in vitro* growth. We therefore employed a protocol established for *in vitro* differentiation of fibroblasts to adipocytes. 786-O cells displayed a modest positive staining with ORO when grown in conventional growth medium. After two weeks of treatment with the adipocyte differentiation medium a strong enhancement of ORO staining was detected (Fig. 4C). To assess the contribution of Notch signaling to the accumulation of lipids we treated the cells with DAPT, an inhibitor of the gamma-secretase activity that is required for activation of endogenous Notch signaling. A pronounced inhibition of the induced lipid accumulation was noted. Together these results suggest that enhanced Notch signaling in the context of ccRCC might contribute the characteristic cytoplasmic lipid accumulation, which is a hallmark of this tumor type.

## Discussion

ccRCC is the most common form of kidney cancer. Worldwide, approximately 270 000 new cases are diagnosed each year, and about 116 000 patients die of the complications of this disease^56^. Metastatic ccRCC is known to be extremely resistant to conventional cancer treatments and the outcome is poor with a median survival of less than one year. For this reason, the development of new therapies that act upon specific abnormalities in ccRCC is highly motivated. In this regard, a mouse model that reflects human ccRCC would offer an excellent opportunity to study and manipulate ccRCC growth mechanisms in a genetically defined setting in the context of ccRCC tumor microenvironment. As loss of *VHL* is so strongly associated with initiation of ccRCC, several efforts have been made to target this tumor suppressor gene in mice. So far, all attempts to generate a ccRCC mouse model that phenocopies the hallmarks of ccRCC, i.e. the clear cell histology associated with perturbed metabolic control in combination with metastatic potential has proven difficult; Mice homozygous for the *Vhl* null allele die *in utero* because of vascular abnormalities of the placenta^57^, while *Vhl*^+/−^ mice^52^ or mice in which *Vhl* was mosaically deleted with a human β-actin driven Cre^58^, did not display any signs of renal pathology. Similarly, conditional inactivation of *Vhl* in the distal tubule and ascending loop of Henle failed to give rise to any renal structural abnormalities^14^,^16^,^17^. Elimination of functional pVHL in the kidney proximal tubule epithelial cells increased the number of renal cysts, but only after long latency and with a low penetrance^15^. Based on these results, it can be concluded that *Vhl* is a rather weak tumor suppressor gene in the kidney and that there is a requirement for additional genetic alterations to provoke ccRCC tumor development. In accordance with this theory, researchers have combined kidney specific deletion of *Vhl* with the deletion of other tumor suppressor genes such as *PTEN*^16^ *p53*^59^ or *Bap1*^60^ The two latter, but not the first, do show signs of renal neoplasms. None of these mice however display aggressive tumor growth or epithelial cytoplasmic lipid accumulation, the latter being a cardinal sign of ccRCC^6^,^7^. Cytoplasmic lipid accumulation was however observed in the renal cortex of mice upon constitutive expression of a stabilized form of the HIF-1α, but not HIF-2α^61^,^62^. These data are somewhat surprising considering the observations indicating that HIF-2α rather than HIF-1α is the main tumorigenic driver in the context of *VHL* deficient tumors. Indeed, tumor suppression by pVHL can be opposed by α overexpression of HIF-2α but not by HIF-1α^11^,^12^.

With regards to the shortcomings of the mouse models based on targeting *Vhl* it can be speculated that it relates to the selection of the Cre-driver genes, which in previous studies are expressed mainly in other tubular compartments than the proximal tubules alternatively already in the early developing nephron^60^. The proximal tubule specific expression and the fact that the *Kap2-iCre* is an inducible system makes it superior compared to the previously used proximal tubular specific promoter^15^, and would thus give a more disease-relevant model system.

The oncogenic role of aberrant Notch signaling has been established in several tumors including T-cell Acute Lymphoid Leukemia (T-ALL)^63^, brain tumors^29^, melanoma^28^, lung carcinoma^26^, pancreatic cancer^64^, and epithelial tumors such as breast cancer^65^,^66^,^67^. *In vitro* and *in vivo* data indicate that activation of the Notch pathway contributes to the malignant features of ccRCC^43^,^44^,^45^,^48^,^49^. In the present study we used the *Kap2* promoter as a driver for an inducible Cre recombinase (Fig. 2A). The expression of the *Kap2* promoter has previously been reported to be limited to the proximal tubules of the renal cortex^53^,^68^. This was verified in our study by crossing the *Kap2-iCre* mouse with the *R26R-YFP* reporter mouse (Fig. 2B) and further by co-staining of the proximal marker LTA with CaIX as a marker for *Vhl* loss.

In contrast to previous model systems based on excision of *Vhl* in the kidney^15^,^60^ we did not note any cyst formation did not occur in our model system. This might be due to the high specificity of the Cre driver to the proximal tubule cells in combination with the pronounced focal expression of the Cre recombinase, as shown by a speckled YFP staining pattern of the proximal tubules in in kidneys of the *Kap2-iCre*/*R26R-YFP* mice. In addition, it should be noted that previous model systems relies on constitutive Cre drivers that are expressed also during nephrogenesis. Thus, it remains possible that the timing of the *Vhl* excision is important for the cystic phenotype. It would therefore be of importance to monitor the *Vhl*^*fl/fl*^/*CALSL-NICD/Kap2-iCre* for time periods that exceed 12 months.

Interestingly, despite the lack of cysts we do observed clusters of dysplastic cells with a clear cytoplasm together with distorted tubules and slightly enlarged nuclei. As indicated by ORO staining these cytoplasms shows an excess accumulation of lipids, a feature not noticeable in normal renal epithelium neither in previous *Vhl* deleted mouse models^14^,^15^,^16^,^17^. This phenotype is significantly more pronounced in proximal tubular epithelial cells in the androgen induced *Vhl*^*fl/fl*^/*CALSL-NICD/Kap2-iCre* than *Vhl*^*fl/fl*^*/Kap2-iCre* mice. Thus, Notch signaling seems to contribute to the cytoplasmic lipid accumulation seen in ccRCC. This notion was further supported by our *in vitro* studies, showing that lipid accumulation in established ccRCC cells induced with using an adipocyte differentiation protocol display less lipid accumulation upon inhibition of Notch signaling. In accordance with our results, it was recently shown that Notch signaling can induce a lipogenesis and lipid storage in hepatocytes^69^.

In our model system, where we combined deletion of *Vhl* with ectopic expression of NICD1, we observed several dysplastic nests of cells with a clear cell phenotype, but only one overt neoplasm. It should be kept in mind that the kidney has a low intrinsic cell turn over. Thus, the promising features of our model system might become apparent upon kidney challenge that induce a regenerative process that might convert the dysplastic cells with clear-cell morphology into a full blown ccRCC. This would also allow for further clarification of the role of elevated Notch signaling during the initiation of renal malignancies.

## Material and Methods

### Generation of transgenic mice

*R26R-YFP*^70^, *Vhl*^*fl/fl* 52^, *CALSL-NICD*^51^ and *Kap2-iCre*^53^ mice strains were purchased from the Jackson Laboratory (Bar Harbor, ME) and were kept under pathogen free conditions. The mice were crossed to generate *R26RYFP*^*tg*/+^*/Kap2-iCre*^*tg*/+^, *R26RYFP*^*tg*/+^, *Vhl*^*fl/fl*^/*CALSL-NICD*^*tg*/+^/*Kap2-iCre*^*tg*/+^, *Vhl*^*fl/fl*^/*Kap2-iCre* and *Vhl*^*fl/fl*^/*CALSL-NICD*^*tg*/+^; mice and were maintained on a mixed C57BL6/129S background. Various genotypes of the offspring were verified by genotyping. List of primers are provided in Supplemental Table 1. To activate expression/excision of genes, 6–8 week old mice were subjected to testosterone treatment by subcutaneous implantation of a testosterone pellet (10 mg testosterone (Sigma Aldrich, Saint Louis, MO) in beeswax). The mice were followed for up to 12 months post implantation. The mice were euthanized and kidneys were put in RNAlater (Life Technologies, Carlsbad, CA) or formalin for further analysis. All animal procedures were approved by the Malmö-Lund Ethical Committee (Dnr. M64-11 and M12-12) and the use of laboratory animals were conducted in accordance with European Union directive on the subject of animal rights.

### Immunostaining

For histological analysis sections of formalin-fixed, paraffin-embedded (FFPE) mouse renal tissue (4 μm) were deparaffinized using xylene followed by graded ethanols for rehydration and stained with hematoxylin and eosin, Periodic-acid schiff (PAS) or Masson’s trichrome according to standard protocol (HistoLab Products AB, Göteborg, Sweden). For IHC boiling in 10 mmol/L citrate buffer at pH 6 was performed as antigen retrieval. Staining was detected using the EnVision system and DAKO Techmate 500 staining equipment according to the instructions of the manufacturer (DAKO, Carpenteria, CA). Paraffin sections were incubated with the following primary antibodies GFP (A290, Abcam, Cambridge, UK), CaIX (AF2344, R&D Systems, Minneapolis, MN), Glut1 (07–1401, Merek Millipore, Darmstadt, Germany), Ki67 (SP6 RM-9601-S, Thermo Scientific, Waltham, MA) and Podocalyxin (AF1556, R&D Systems, Minneapolis, MN). For Oil red O (ORO) stain kidneys were fixed in formalin over night followed by cryo preservation in 30% sucrose over night followed by embedding in optimal cutting temperature compound (O.C.T.) and sectioned (10 μm) on a cryostat.

### Lectin and immunoflourescent staining

FFPE mouse renal (4 μm) sections were deparaffinized using xylene followed by graded ethanols for rehydration according to the standard protocol. Antigen retrieval was performed using Diva Decloaker solution (Biocare Medical, Concord, CA). After being blocked with blocking buffer containing 5% BSA in PBS-T, the sections were incubated over night, 4 °C with Lotus tetragonolobus agglutinin (LTA) (FL-1321, Vector laboratories, Burlingame, CA) and CaIX (AF2344, R&D Systems, Minneapolis, MN). After washing, secondary antibody donkey -anti rabbit AlexaFluor-594 (A-11056, Life technologies, Carlsbad, CA) and nuclear stain To-pro-3 iodide (Life technologies, Carlsbad, CA) were applied according to the manufacturer’s instructions followed by rinsing and mounting. Sections were subsequently analyzed by confocal scanning using the Zeiss LSM 710 system (Carl Zeiss AG, Oberkochen, Germany).

### Acquisition of human Renal Tissue

Renal tissue for tissue microarray (TMA) was obtained from nephrectomies performed owing to ccRCC malignancy (n = 57) or from renal biopsies (n = 4) performed using 18-gauge needles. All the specimens were collected after informed consent was obtained from the patients. Ethical permission was granted by the ethical committee at Lund University (D282–07) and all experiments were carried out in accordance with the approved guidelines. The TMA was stained with Notch 1 antibody (3E12, Sigma Aldrich Saint Louis, MO).

### Real-Time Quantitative PCR

Tissue was collected in RNAlater (Life Technologies, Carlsbad, CA) followed by homogenization using TissueLyser LT (Qiagen, Hilden, Germany). Total RNA was extracted in using RNeasy Mini Kit together with the Qiashredder Kit (Qiagen, Hilden, Germany). An on-column DNAse treatment was included during the extraction. cDNA synthesis was performed using random primers and MultiScribe Reverse Transcriptase enzyme (Applied Biosystems, Foster City, CA). The amplifications were run using 7300 Real-Time PCR (RT-PCR) System (Applied Biosystems, Foster City, CA) with SYBR Green Master Mix (Applied Biosystems, Foster City, CA) and all the detections were performed in triplicate. The housekeeping gene *Gapdh* was used for normalization. Comparative CT method was used to quantify relative mRNA. The following primer sequences were used: *Gapdh* forward 5′-TCGTGGATCTGACGTGCCGCC-3′; *Gapdh* reverse 5′-CACCACCCTGTTGCTGTAGCC-3′; *Notch1* forward 5′-CGGGTCCACCAGTTTGAATG-3′; *Notch1* reverse 5′-GTTGTATTGGTTCGGCACCAT-3′; *Hey1* forward *5*′*-*CTTGAGTTCGGCTCTGTGTTCC-3′′; and *Hey1* reverse 5′-GATGCCTCTCCGTCTTTTCCT-3′.

### Cell culture and adipocyte differentiation of 786–0 cells

786–0 cells (ATCC, Rockville, MD, USA) were maintained at subconfluence in DMEM High Glucose (GIBCO, Invitrogen, Carlsbad, CA) supplemented with 10% FBS and 1% PEST. Cells were seeded in 6-well plates on coverslips and two-days postconfluent cells were treated 48 h with 0.1 uM dexamethasone (Sigma Aldrich, Saint Louis, MO), 10 ug/ml insulin (Sigma Aldrich Saint, Louis, MO), 100 uM indomethacin (Sigma Aldrich, Saint Louis, MO), 500 uM IBMX (Sigma Aldrich, Saint Louis, MO) and 10 uM DAPT (Sigma Aldrich, Saint Louis, MO) or DMSO followed by 12 days of treatment with 10 ug/ml Insulin (Sigma Aldrich, Saint Louis, MO) and 10 uM DAPT (Sigma Aldrich, Saint Louis, MO) or DMSO with medium change every second day. Coverslips were washed in PBS and fixed in PFA, stained with Oil Red O and counterstained with Mayer’s hematoxylin according to standard procedures, followed by mounting in Faramount aqueous mounting media (DAKO, Carpenteria, CA). Imaging was performed using Olympus BX63 microscope equipped with UPlanSApo objectives (10x/0.40), (20x/0.75), (40x/0.95) and DP80 camera using the CellSens Dimensions imaging software. Images are representative from triplicate experiments.

### Bioinformatics analysis

Level 3 RNA-seq data containing mRNA gene-level RSEM estimates were downloaded from The Cancer Genome Atlas (TCGA) data portal (http://tcga-data.nci.nih.gov/tcga/dataAccessMatrix.htm) by September 2014. Gene expression levels for each sample were created by multiplying the gene level RSEM estimate values by 10^6^ followed by adding an offset of 1 and subsequent log2 transformation. The data set comprised 530 primary clear cell renal carcinomas and 70 normal kidney tissue samples.

### Statistical analysis

If not otherwise stated, data is presented as mean ± standard deviation. For statistical analysis unpaired t-test was used. P- values: *p < 0.05, **p < 0.01, ***p < 0.001.

## Additional Information

**How to cite this article**: Johansson, E. *et al.* Simultaneous targeted activation of Notch1 and *VHL*-disruption in the kidney proximal epithelial tubular cells in mice. *Sci. Rep.* **6**, 30739; doi: 10.1038/srep30739 (2016).

## Supplementary Material

Supplementary Information
