# Supplementary material for: Simultaneous targeted activation of Notch1 and Vhl-disruption in the kidney proximal epithelial tubular cells in mice
Source: Sci Rep. 2016 Aug 5;6:30739. doi: 10.1038/srep30739 (PMC4974510; doi:10.1038/srep30739)

Supplemental Material: Simultaneous targeted activation of Notch1 and VHL-disruption in the kidney proximal epithelial tubular cells in mice.

E. Johansson, B. Rönö, M. Johansson, D. Lindgren, C. Möller, H. Axelson, E. Smith

Supplemental Table 1. Primers for genotyping

Strain	Primers for genotyping
Vhl	2-lox-VHL forward-5´-CTGGTACCCACGAAACTGTC-3´<br>2-lox-VHL reverse-5´-CTGACTTCCACTGATGCTTGTCACAG-3´<br>1-lox-VHL forward-5´-CTAGGCACCGAGCTTAGAGGTTTGCG-3´<br>1-lox-VHL reverse-5´-CTGACTTCCACTGATGCTTGTCACAG-3´
Nicd	CALSL-NICD forward-5´-CAACATCCAGGACAACATGG-3´<br>CALSL-NICD reverse-5´-GGACTTGCCCAGGTCATCTA-3´<br>CALSL-NICD internal ctrl forward-5´-CTAGGCCACAGAATTGAAAGATCT-3´<br>CALSL-NICD internal ctrl reverse-5´-GTAGGTGGAAATTCTAGCATCATCC-3´
R-YFP	RYFP-1 mutant 5´-AAGACCGCGAAGAGTTTGTC-3´<br>RYFP-2 common 5´-AAAGTCGCTCTGAGTTGTTAT-3´<br>RYFP-3 wt 5´-GGAGCGGGAGAAATGGATATG-3´
iCre	icre forward 5´-GGCCTTTGAACGCACTGAC-3´<br>icre reverse 5´-AGGGGCAGCCACACCAT-3´

Supplemental figure legends

Figure S1.

FFPE kidney sections from *Vhl*$^{fl/fl}$*/CALSL-NICD/Kap2-iCre,* *Vhl*$^{fl/fl}$*/Kap2-iCre* and *Vhl*$^{fl/fl}$*/CALSL-NICD* mice 12 months after the onset of androgen treatment stained with (a) an antibody raised against the proliferative marker ki67, (b) Masson´s Trichrome stain, (c) and an antibody raised against podocalyxin. Scale bars, 100 µm.

Supplemental figure 1

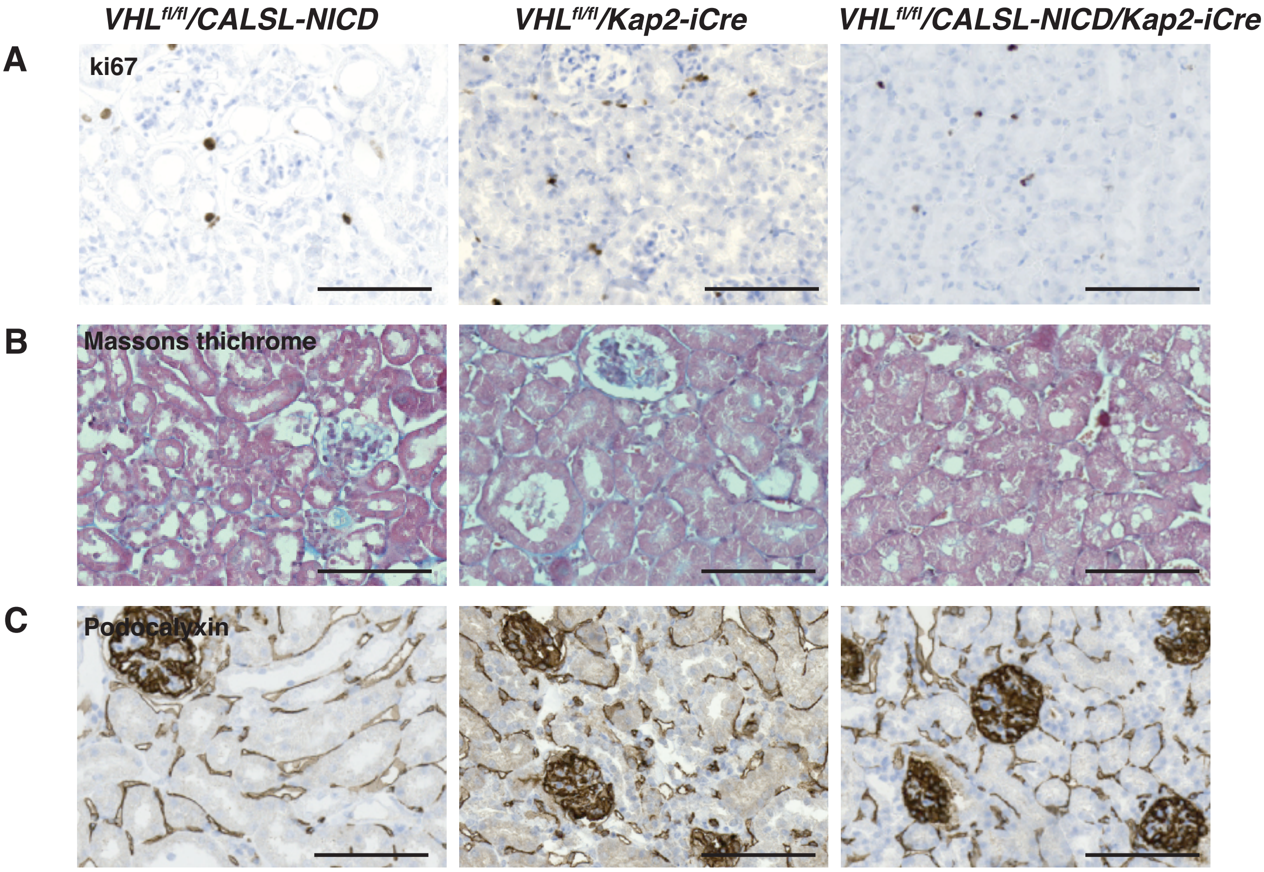

Supplement: Supplementary Information [file srep30739-s1.pdf]
